# A Literature Review Study on Atomic Ions Dissolution of Titanium and Its Alloys in Implant Dentistry

**DOI:** 10.3390/ma12030368

**Published:** 2019-01-24

**Authors:** Sammy Noumbissi, Antonio Scarano, Saurabh Gupta

**Affiliations:** 1 International Academy of Ceramic Implantology, Silver Spring, MD 20910, USA; sammy@iaoci.com (S.N.); saurabh@iaoci.com (S.G.); 2 Department of Medical, Oral and Biotechnological Sciences and CeSi Met, University of Chieti-Pescara, 66100 Chieti, Italy; 3 Zirconia Implant Research Group, Silver Spring, MD 20910, USA; 4 Oral & Maxillofacial Surgeon and Implantologist, Private Practice Dentistry, Bangalore 560042, India

**Keywords:** corrosion of titanium implants, titanium corrosion, metal ion release from the titanium implants, fretting and pitting corrosion, implant corrosion, peri implantitis and corrosion, crestal bone resorption

## Abstract

This review of literature paper was done in order to conduct a review of the literature and an assessment of the effects of titanium implant corrosion on peri-implant health and success in the oral environment. This paper evaluates and critically reviews the findings of the multiple in-depth in vivo and in vitro studies that are related to corrosion aspects of the titanium and its alloys. A literature survey was conducted by electronic search in Medline and studies that were published between 1940 and August 2018 were selected. The search terms used were types of corrosion, corrosion of titanium implants, titanium corrosion, metal ion release from the titanium implants, fretting and pitting corrosion, implant corrosion, peri implantitis, and corrosion. Both in vivo and in vitro studies were also included in the review. The search and selection resulted in 64 articles. These articles were divided on the basis of their context to different kinds of corrosion related to titanium dental implants. It is evident that metal ions are released from titanium and titanium alloy dental implants as a result of corrosion. Corrosion of implants is multifactorial, including electrical, chemical, and mechanical factors, which have an effect on the peri-implant tissues and microbiota. The literature surveyed showed that corrosion related to titanium and its alloys has an effect on the health of peri-implant soft and hard tissue and the long term survival of metal dental implants. It can be concluded that presence of the long-term corrosion reaction along with continuous corrosion leads to the release of ions into the peri-implant tissue but also to a disintegration of the implant that contribute to material fatigue and even fracture of the abutments and implant body or both. This combined impact of the corrosion, bacterial activity, chemical reactions, and functional stresses are to be looked at as important factors of implant failure. The findings can be used to explore the possible strategies of research to investigate the biological impact of implant materials.

## 1. Introduction

The gateway to human body is the mouth. It is the habitat for a wide variety of microorganisms and with saliva maintaining its wetness makes it a very harsh environment for dental materials and implants. Oral tissues are exposed not only to chemical and physical attack, but also to metabolic action of nearly 30 species of the bacteria of oral microbiome [1].

The real count of bacteria is calculated to be nearly more than one billion per mL of the saliva, however a biological equilibrium takes place and the oral tissues remain healthy for the most part. Saliva is host to a variety of viruses, yeast, bacteria, and fungi, along with their byproducts, enzymes, food debris, organic acids, components of crevicular fluids, and epithelial cells. In addition, saliva is a hypotonic solution with a pH level ranging from 5.2–7.8 and it contains potassium, sodium, nitrogen, chloride, bio-actonate products, and proteins. The major part of dental plaque near the teeth is constituted of both gram-negative and gram-positive bacterial species, which also colonize the mucosal surfaces [2,3].

Until recently, noble alloys were considered to be chemically inert in the body and in the oral environment in particular. Therefore, they were not considered to be vulnerable or susceptible to corrosion attack in the oral environment. Corrosion can be characterized by gradual degradation of materials by processes of the electrochemical events. The bio-compatibility for the metal and metal alloy dental implants is based on the material ability to be immune to corrosion attack, its mechanical properties, and response to host factors. The most commonly used components in implant alloys are titanium, vanadium, and aluminum. Corrosion greatly compromises the strength and the fatigue resistance of metal alloys and it will lead to structural and mechanical failure of dental implants and their prosthetic components. The corrosive phenomena of titanium implant increase the possible deterioration of the mechanical properties of the dental implant components and mechanical tests confirmed that the strength of the dental systems subjected to masticatory loads are strictly related to the bond at the interface [4,5]. This is of concern mainly when the implants fixtures and their prosthetic components are coupled in the human mouth, which is a known electrolytic and hostile environment. The release of the metal ions as a result of the corrosion has greatly proven to be the factor in osteolysis and peri-implantitis around the metal implants [6,7].

## 2. The Clinical Relevance of Corrosion

Even though titanium alloys are extremely corrosion resistant thanks to the stability of the titanium oxide layer, the alloys remain susceptible to corrosion attack. Corrosion is an electrochemical phenomenon and the types of corrosion observed in the oral cavity are in the category of wet corrosion, because the oral cavity is a humid environment. The clinical manifestations of dental implant materials corrosion range from soft tissue discoloration to bone loss. Corrosion causes release of metal ions to the surrounding hard and soft tissues, lymph nodes, peripheral, and even distant organs. The metal ions may cause staining of the peri-implant tissues and reactions, like perioral stomatitis, osteolysis, oral edema, and extra-oral manifestations, such as fatigue, hair loss, eczematous rashes, and even episodes of brain-fog have been reported. Kirkpatrick et al. reported the patho-mechanism of the impaired healing occurs due to specific metal ion release by corrosion [8].

### 2.1. Types of Corrosion in the Oral Cavity

The wet corrosion reactions with dental implants are of three types: mechanical, electrochemical, and chemical. The concentration in certain components of saliva, pH, buffering capacity, and surface tension all play a role in its properties as an electrolyte. Therefore, the magnitude of the net process of corrosion is affected by multiple variables.

**Figure 1:**
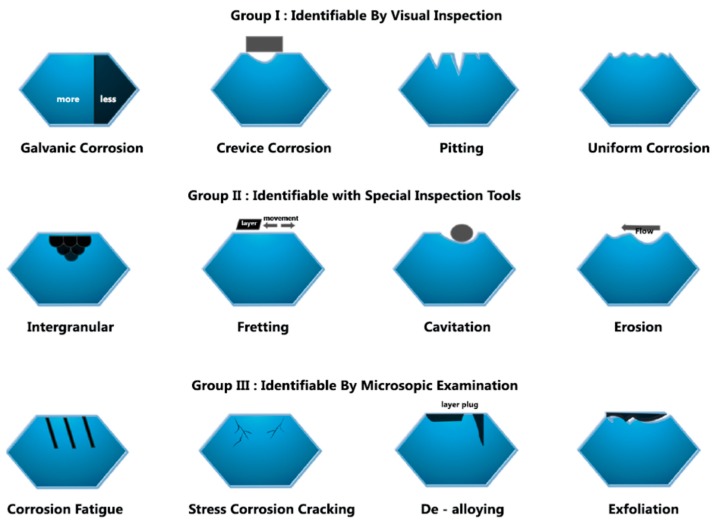
Diagrammatic presentation of different forms of corrosion.

The wet/electrochemical corrosion requires an electrolyte or wet environment. Mechanical corrosion is a result of the functional stresses on the implant and its prosthetic components, resulting in scratching, weakening, and cracking of the components, ultimately making them vulnerable to corrosion attack [9]. These are the types of corrosion that takes place in oral cavity (Figure 1).

Intra-oral corrosion types and their definitions:

Uniform Corrosion—It is a regular and uniform removal of metal ions from the implant or implant prosthetic superstructure surface.

Pitting Corrosion—It is a localized form of the corrosion occurring on openly exposed metal surfaces in the absence of any apparent crevices. This occurs usually along with fluoride based solutions used during dental procedures and home care.

Crevice Corrosion—It occurs in the constricted or in between two close surfaces where there is no exchange of oxygen. This occurs mainly at the implant-abutment interface.

Galvanic Corrosion—This type of corrosion takes place where the dissimilar types of alloys are in direct contact with each other within the oral cavity. Metal ions are therefore released as a result of galvanic activity. In such cases, the implant plays the role of an anode.

Stress Corrosion—This occurs when metal fatigue occurs under function, cracks form, and the implant becomes vulnerable to corrosion when in contact with the corrosive environment.

Erosion and Fretting Corrosion—This type of corrosion take place between two metals that are in contact and rub against each other during function. This occurs at contact surfaces, such as at the abutment-implant interface.

Microbial Corrosion—The inflammatory processes as a result of host response to the materials and the by products released by anaerobic bacteria create an acidic environment that is corrosive to the implant and its prosthetic parts.

**Figure 2:**
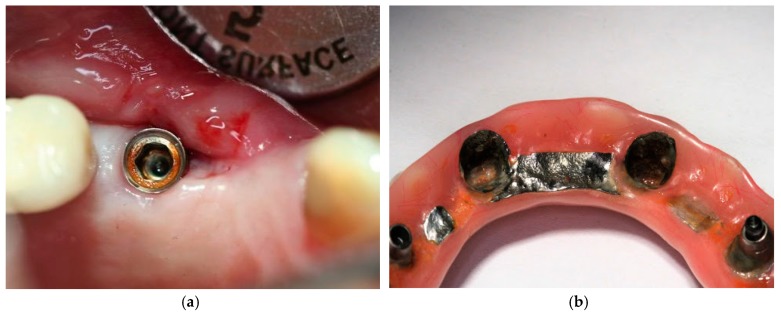
(**a**) Corroded implant-abutment connection; and, (**b**) Corroded superstructure hybrid denture.

The most common and easily clinically observable types of corrosion are crevice, galvanic, and pitting corrosion. It should however be understood that the different corrosion types occur independent of each other but at the same time, and in some instances complementary to each other or an extension of an earlier type of corrosion. The intricacy of the underlying electrochemical processes between the superstructure and the implant is associated with phenomenon of pitted corrosion, which is a result of galvanic coupling. Galvanic corrosion itself occurs as a result of ion exchange between implants and their prosthetic components. Pitted corrosion happens often at connection of implant and abutment (Figure 2a,b). Crevice corrosion propagates and occurs because of the increased concentration of chloride ions and reduction of the pH value thus creating an acidic environment. With increased acidity of the peri-implant area, the passive oxide layer that was once formed as a result of exposure to oxygen at the time of implant placement dissolves and leaves the implant susceptible to corrosion attack [10]. The flow of oxygen that is necessary to repassivate the titanium surface is compromised the combination of crevice and acidic environment [11]. In fact, the Streptococci adversely lower the electrochemical behavior of titanium surfaces [12].

#### 2.1.1. Galvanic Corrosion

This is the most common type of corrosion found with dental implants. Gold alloys are selected for the prosthetic superstructures because of their excellent capacity of corrosion resistance, mechanical properties, and biocompatibility [10,13,14]. However, they are very expensive and new alloys, such as Co-Cr, Ag-Pd, and Ni-Cr are commonly used. When prosthetics parts or dental restorations that are made from the dissimilar alloys come in contact with each other and are coupled with the implant while exposed to oral fluids, the difference in potential creates electric current flow, also called galvanic currents and cells. Also, saliva and fluid penetrates into the inner of implants through the micro-gap between implant and abutment [15], generates currents with the metal dissolution, due to a potential difference created by the formation of a galvanic cell [16]. These electrical currents lead to the acceleration of the corrosion rate of the lesser noble metal. Galvanic currents pass through metal-to-metal junction and also through tissues that flow via electrolytes, like tissue fluids and salvia. The risk of corrosion as a result of galvanic coupling has thus become elevated as a result of the increased amounts of alloy components. The often coupling of implants with dissimilar metal alloy prosthetic components, such as abutments and abutment screws, must be of concern, especially when selecting the materials that are used in superstructures on implants.

#### 2.1.2. Pitting Corrosion

Pitting and stress corrosion occur at the surface of the prosthetic components and implants as a result of stress and function. This occurs as a result of small pits and crevices that make both the implant and the prosthetic structures vulnerable to the corrosion attack and thus become sites where pit and stress corrosion originate. Some of the other types of corrosion that are either closely related to or occur in concert with pitting corrosion are stress corrosion, torsional, and notched material fatigue corrosion [17].

#### 2.1.3. Fretting Corrosion

The joint action of chemical and mechanical attack leads to fretting corrosion. It occurs around the restored implants in the oral cavity. The hydrogen attack happens when the hydrogen reacts with the content of carbide in the steel, which forms methane. This results in surface blisters and decarburization voids, which are areas vulnerable to corrosion attack [18].

#### 2.1.4. Microbial Corrosion

Corrosion that is associated with microbiology has been well investigated and demonstrated in the literature. It has also been observed that the microorganisms lead to the corrosion of metal alloys when they are immersed in aqueous conditions. Loading forces on the crown under function may cause the bending of the components or movements inside the implant abutment, with the formation of a micro or larger gaps and with access of bacteria and fluids [19,20]. According to Chang et al., the corrosion of metal dental alloy materials increases in the presence of the streptococcus mutans, where the byproducts trigger an inflammatory process that renders the peri-implant environment acidic and corrosive [21]. The attachment and accumulation of the bacteria on the implant surfaces disrupt the passivity of implant alloys and the organic acids formed at the time of glycolysis of sugars reduces pH. All of these biological events make the peri-implant environment acidic and provide an environment conducive to corrosion with the participation of anaerobic bacteria. Bacteria and other microbes oxidize manganese and iron and products such as MnO_2_, FeCl_2_, MnCl_2_, Fe_2_O_3_, and FeO that are favorable for corrosion. The interaction of the anaerobic and aerobic bacteria is a complex mechanism in various zones that favor the corrosion process. With biofilm build up, the metal surface underneath the biofilm is exposed to different levels of oxygen concentrations, which leads to the formation of the differential aeration cells. The less aerated zones play the role of anode, which undergo corrosion and the release metallic ions in saliva and peri-implant tissues. These metallic ions join the end yields of the bacteria and chloride in saliva forming corrosive products, such as MnCl2 and FeCl2, which cause further corrosion. These phenomena of acidic waste products of bacteria and microbes synergistically contributing to the corrosion of metal surfaces are called microbial corrosion [7,22].

### 2.2. The Impact of the Corrosion on Dental Implants

**Figure 3:**
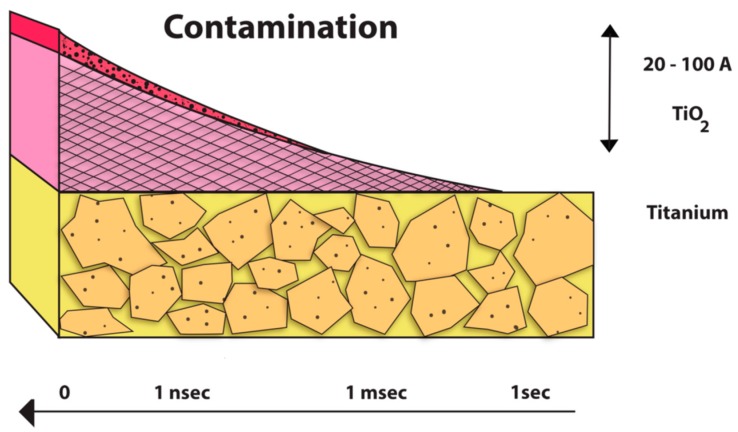
Graphical representation of TiO_2_ layer depletion.

At present, the majority of dental implant systems are alloyed and generally made of pure titanium or Ti alloy (Ti-6Al-4V) of which aluminum is a base metal. The titanium and its alloys provide improved physical properties along with good resistance against corrosion in acidic and saline environments. Although titanium alloys highly resist the corrosion because of the stability of the oxide layer TiO_2_, they are known to be vulnerable to the corrosion attack, especially when the inert layer of titanium oxide is breached or removed and not able to reconstitute itself. The result is an oxidation process with the release of corrosion products and metal ions to the peri-implant hard and soft tissue (Figure 3). There is a formation of an electrochemical cell if the prosthetic parts have less noble or base alloy components. The corrosion occurs with formation of anode by the lesser noble metal alloy and cathode forms by the more noble material, which is the high titanium content alloy implant. The electrons are transferred to the anode, which becomes the surface where the positive ions get accumulated. The resulting effect is that the implant surface undergoes oxidation, corrodes, and releases ions. The metallic ions can also be released when base alloy superstructures are connected to the implants during delivery of the permanent restoration [23].

#### 2.2.1. Dental Implant- Fracture

Fractures of the dental implants are an uncommon incidence that results in unfavorable clinical outcomes. Corrosion compromises the fatigue life and strength of a material and make implants susceptible to mechanical failure. When in function and subjected to the intermittent loads, titanium alloys undergo wear and tear and with time become vulnerable to corrosion. On other hand, in immobile situations, titanium and its alloys are static and they can withstand physiologic chlorine solution at body temperature [24,25,26]. However, they are vulnerable to the oxide changes that are produced by mechanical micro motion. For example, titanium alloys display crack-like failures while under functional loading stress. Thus, repeated breakdown of the oxide layer may undermine corrosion resistance. After the corrosion starts, the ions leak and get released to the surrounding tissues that lead to complications ranging from mild local discoloration to implant fracture and/or failure. In a study by Green et al., the fracture of the dental implant was reported after 4 years of loading. The failure analysis of the implant presented the fracture was caused due to metal fatigue and corrosion occurred due to the crown metal alloy made of Ni-Cr-Mo [24]. Yokoyama, et al. concluded that in the biological environment, the titanium absorbed hydrogen, was the reason for the titanium implant delayed fracture [25].

#### 2.2.2. Cellular Responses

In cell cultures, titanium ions influence phenotype and cell function of T lymphocytes, induce differentiation of monocytes into active osteoclasts, and enhance the expression of cytokines in osteoclasts. The ions of hexavalent chromium are released from the implant materials [27]. Nickel and Chromium cause type IV hypersensitivity [28]. They act as haptens, mutagens, and carcinogens that can cause various cytotoxic reactions involving a decrease of some enzyme activities, increasing mutagenicity, interfering with the biochemical pathways, and increasing carcinogenicity. The titanium implant alloys or the superstructure that include nickel, even in very little amounts, may be the reason for the localized tissue irritation, as observed in some patients and long-term exposure of the nickel in the dental materials adversely affects oral mucosal cells and human monocytes. The manganese also present in the alloys is taken in with saliva that produces toxicity, which leads to skeletal, systemic, and nervous disorders [29]. Lechner et al. clinically explained the possible interrelation between titanium implants and fatty degenerative osteonecrosis as a cause of silent inflammation [30].

**Figure 4:**
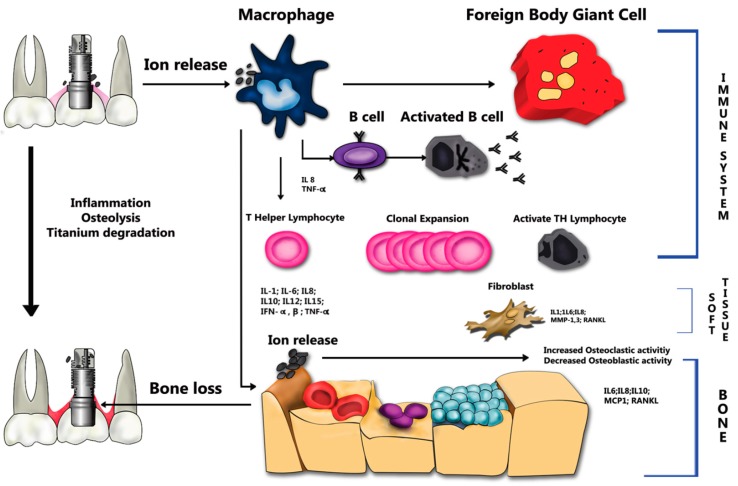
Schematics of titanium degradation process and ion release.

Clinically, the release of ions from titanium-based alloys stimulates the attraction of T lymphocytes and macrophages from the immune mechanism [1,31,32,33,34]. Noronha et al. determined that the quantity and physicochemical properties of the degradation products would determine the magnitude of the effect on peri-implant tissues [35]. High levels of inflammatory mediators involved in peri-implant disease and bone resorption have also been associated to ion release, as illustrated in Figure 4.

Atomic ions and titanium particles are insoluble in physiological medium that results in huge storage and minimal content in the urine. However, aluminium and vanadium ions are more soluble, thus having a cytotoxic effect [36]. Presently, Ti6Al4V alloys (Grade -V) is used to manufacture the most commercially available titanium implants and abutments, which can release aluminium and vanadium atomic ions to adjacent tissues during wear and corrosion processes [17,37,38].

Titanium and its alloys have shown bone and soft tissue integration. However, titanium alloys have certain components, which may affect osseointegration, mainly caused by the corrosion products release from the implant and/or its prosthetic components that contain aluminum and vanadium products. It was presented by Roynesdal et al. that the loss of marginal bone surrounding the implants was more significant in cases where implants were sprayed with TiO_2_ [39]. The disintegration of the hydroxyapatite coating from implant surface results in the exposure of the metal and corrosion attack available. This eventually leads to the release of metal ions and granular debris that cause osteolysis, peri-implantitis, and implant failure. According to Olmedo et al., the presence of macrophages in peri-implant soft tissues, which is affected by the corrosion process, plays a vital role in the implant failure [18]. The free ions of the titanium induce biological events and reactions that cause a loss of biological stability and local osteolysis around the dental implant. This is to be considered or investigated as a potential reason of the peri-implantitis. Recent studies have revealed that the increased level of the dissolved titanium was consistently associated with implants that are affected with peri-implantitis. The presence of titanium particles has been found to modify the peri-implant microbiome structure. It induces epigenetic changes via DNA methylation leading to a modification of gene expression in the DNA of the microbiota, thereby increasing the risk and incidence of inflammatory disease [1,32].

Even though titanium resists corrosion, in past years it has been shown to react to microbiota, living tissues and lead to the physiologic changes in the peri-implant tissues. High quantities of calcium and phosphorus have been found in the oxide layer at the surface of the implants, which confirms the exchange of ions. The ions released as a result of corrosion cause inflammation and local pain in peri-implant tissues that can result in aseptic implant failure and secondary infection [34,40].

#### 2.2.3. Fluoride & Implant Corrosion

**Figure 5:**
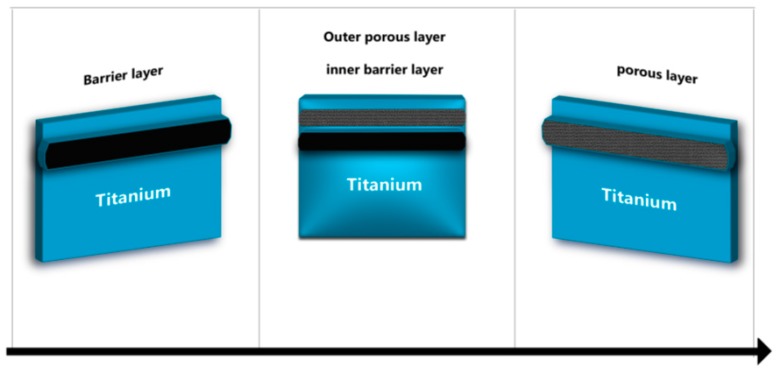
Effect of flouride on corrosion.

The fluoride in commercial mouthwashes, gels, and toothpaste are generally used to prevent dental caries or relieve dental sensitivity. The degradation by the fluoride ions on corrosion resistance of the titanium and its alloys are reported to be extensive. The fluoride ions are particularly aggressive on the protective oxide layer, which forms on the Ti and its alloys. Thus, the odontogenic fluoride gel should be avoided because they produce an acidic environment that leads to the degradation of the TiO_2_ layer and it makes the implant vulnerable to corrosion. It also prevents osseointegration or leads to loss of bone or implant failure. Studies have investigated the effects of topical fluoride on pure titanium and have also determined that toothbrushes that are used in contact with titanium surfaces should be as non-abrasive as possible and contact with topical fluorides should be avoided [41]. The study about the relationship between pH values and fluoride concentrations helped to conclude that fluoride ions have negative effect on corrosion resistance of the pure titanium and its alloys Ti-6Al-4V [40,41,42] (Figure 5).

## 3. Discussion

The primary requisite for choosing metals for intra oral and dental rehabilitation is that no ions must be released as a result of corrosion. Reed and William reported and demonstrated the presence of galvanic currents in the oral cavity as a result of metallic dental restorations [43]. In addition, approximate values for the currents were also set up. Burse et al. described the process for in-vivo tarnish evaluations and proved the significance of suitable elements ratio in the gold alloys [29].

Table 1, shows various experimental studies regarding corrosion. Tufekci et al. described the sensitive analytical technique that showed release of the individual elements in one month that is associated to the microstructural phases in the alloys [44]. In controlled in-vitro experiments, it was concluded that dissolution of titanium alloys is to be considered as the source of the metal ions, which can also lead to toxicity [45]. A study by Rodrigues et al. suggested that acidic environments that are generated by the bacterial biofilms or/and inflammatory processes might lead to titanium surface oxidation of the titanium dental implants [34]. The process of corrosion causes the permanent breakdown of the oxide layer. This, along with the release of metal ions and debris in the peri-implant area, will interfere with re-osseointegration of the implant with bone.

Many biological changes occur as a result of galvanic coupling and they have been widely demonstrated in the literature. The mechanisms of electrochemical kinetics and electrochemical thermodynamics were reviewed to understand corrosion behavior of metals and alloys in the presence of body fluids. Recent findings have proven that higher levels of the titanium ions were detected in the sub-mucosal plaque of the dental implants with peri-implantitis as compared to healthy implant. This indicates correlation in between peri-implantitis and titanium dissolution [31]. In fact, one of the effects of the ionic release on the part of the surface to impregnate could generate changes in the composition of the peri-implant microbiota with very important clinical repercussions on peri-impla health [1], with the activation of the inflammatory response and release of pro-inflammatory cytokines [46,47,48]. Today, scientific evidence shows that there are differences in the microbiota between peri-implantitis and periodontitis [49]. The effects on cell viability are correlated the cytotoxic effect of pH or ion concentrations and fluoride release [50]. Cell cultures are commonly used to evaluate sample biological behavior when in contact with human tissues and cytotoxic effects of metallic ions has been reported in the literature on different cell lines [51]. Moreover the metal ion release may be associated with mutagenic and carcinogenic activity around the dental implant [52]. Galvanic corrosion of Ti along with amalgam and cast alloys was studied in-vitro by Sutow et al. [53] and other studies have shown that, when amalgam was placed in contact with titanium, changes could be seen [54,55]. No changes however were detected in the pH when cobalt, silver palladium, carbon composite, chromium, gold, or stainless steel was put in metallic contact with the titanium. According to Geis-Gerstorfer et al., the galvanic corrosion of implant and superstructure systems is critical in two ways: First, there is a biological impact on the peri-implant soft and hard tissues that result from the dissolution of the alloy and second current flow from the galvanic corrosion lead to bone resorption [16]. According to Reclaru and Mayer, many dental alloys that are used for implant prosthetic superstructures develop some degree of galvanic coupling along with the titanium. They found that titanium underwent accelerated corrosion even in the presence of precious alloys [56]. Cortada et al. reported similar findings that metallic ions were released from the titanium implants that were coupled with several metal superstructures made from machined titanium, silver-palladium, chrome-nickel, gold, and cast titanium alloys in artificial saliva. In addition, it was also determined that the ions are released from the implants with have superstructures made from of dissimilar metal alloys [27].

A study by Aparicio et al. characterized the corrosion of pure titanium shot blast with many materials of different sizes of the shot particles in the dental application [61]. The roughness of surface is increased when the metal is blasted. This roughness is added with bone to improve implant fixation. This demonstrates increased surface area of material due to increased surface roughness. The differences can be identified between corrosion resistance and the electrochemical behavior of the pure Ti after shot is blasted for enhanced osseointegration and roughness. Oh and Kim carried out a study about electrochemical properties contained in superstructure was coupled galvanic ally to the titanium implant [62]. The photo-micrographs were taken after testing electrochemical displayed crevice or pitting corrosion at marginal gap and at the surface of superstructure. Samples of the Ti/Co-Cr implant combination revealed possible galvanic corrosion. Another study also revealed the dissolution of corrosion products in adjacent tissues and bio liquids. To conclude, it can be said that monitoring of the corrosion potential indicates existence of and also the degree of galvanic and other kinds of the corrosion in the titanium alloy dental implant. Lopez-Alias et al. concluded that the prediction of clinical behavior of alloy from the in-vitro studies is very difficult, because the factors may change because of quality and quantity of saliva, diet, alloy polishing, genetics, oral hygiene, amount, and distribution of the occlusal forces, microbiota, and brushing using toothpaste that influences degree of corrosion [63]. The increased level of the content of metal ion in environment will eventually lead to further corrosion, not only by their mere presence, but also as a result of the inflammatory response that they trigger. Under general circumstances, the corrosion of metal stops when the ions become saturated in the surrounding environment, however this does not occur with dental restoration because the dissolving fluids, like saliva, drinks, toothbrushing, and food remove ions, thereby allowing for a continuous corrosion process.

## 4. Materials and Methods

This study conducts a review of the literature extending from 1940 to February 2018 obtained via electronic search in medline. The search terms used were- corrosion types, corrosion of titanium implants, titanium corrosion, metal ions release from the titanium implants, fretting and pitting corrosion, implant corrosion, implant corrosion, and peri-implantitis. Both in vivo and in vitro studies were also included in the review. 425 articles were selected and 32 articles were removed because they were duplicates; the remaining 393 articles abstracts were read. 344 articles were excluded because they did not investigate dental implant corrosion in vitro or in vivo. Finally, 64 articles were included in this systematic review.

## 5. Conclusions

Titanium remains the predominant material used for oral implants. Despite high strength and good resistance to corrosion, titanium particles, multiple studies have demonstrated and proven that degradation by products of titanium and titanium alloys have been detected in oral tissues and in distant organs. Titanium particles are released from the surfaces of dental implants for many reasons, such as mechanical wear, contact with chemical agents, and bacteria embedded in adherent biofilm and inflammatory cells. Inflammation leads to two types of host response, namely hypersensitivity and toxic/pro-inflammatory. However, there is poor specificity as the observed reactions could be initiated by other factors that are associated with the placement of implants.

In vitro experiments have shown the potential of titanium ions or particles to have toxic or pro-inflammatory effects. In fact, in the inflammatory process of the peri-implant soft tissues sites with the presence of progressive loss presented a higher number of metallic particles when compared to healthy implants [64]. In vitro research has also identified factors modulating such effects, for example, particle size and association with molecules, like lipopolysaccharide. Titanium particles are commonly detected in healthy and diseased peri-implant mucosa alike, and even in gingiva of individuals without titanium implants. Such observations should make us look at the environment as a source of these particles and how they might contribute in the immunologic response that some patients have to dental metals and titanium alloys. Thus, there is poor specificity for the association between presence of particles and pathology. There is a tendency to find more titanium in close proximity of the implant surface and in specimens from diseased sites. However, higher concentrations of titanium in diseased sites could be the consequence of corrosion caused by the activity of inflammatory cells and bacteria that are present in peri-implant lesions.

There is some biological plausibility for a link between corrosion, the presence of titanium particles, and biological responses that ensue. However, proof for a unidirectional sequence of causative events does not exist. Wear, corrosion, titanium particles, inflammation, and microorganisms take part in a complex host response to foreign bodies with multiple feedback loops: Wear and corrosion together with environmental factors lead to material degradation in a process called tribocorrosion; this process leads to the release of titanium particles. Titanium particles interfere with cell function and they promote an inflammatory cascade that causes corrosion, and also alters the composition and function of the biofilm. Ultimately, biofilms cause inflammation and contribute to the complex phenomenon of corrosion.

It must be mentioned that there are also observations that are not in line with this hypothesis, because metal oxide nanoparticles, especially of TiO_2_, possess antimicrobial activity; nano-antibiotics use metal oxides to carry out their functions and they are currently in advanced stages of development. Despite the fact that all currently available protocols for therapy of mucositis and peri-implantitis further contaminate the peri-implant tissues with titanium particles, they have had a certain degree of success.

Given the widespread use of titanium and its alloys in dental implantology, corrosion-induced implant failures will most likely rise and still there remain many grey areas with regards to the biology and physiology on the interaction of dental implant alloys and the host. The outcome from this systematic review shows that Ti particles are a common finding around the dental implants. Hence, continued research and investigations need to continue.

## Figures and Tables

**Table 1:** Presenting some of the experimental studies on corrosion of the dental implants.

References	Implant Alloys	Medium, Temperature, PH, Period, Method, etc.	Remark
Tomofumi Sawada et al. [57].	“Alloys of cast titanium having 20 mass percent chromium or Ti-20Cr & pure titanium”	Electrochemical corrosion at the 0.3 V in 0.9 percent saline solution & mechanical damage that uses ten scratching cycles with 3 various scratching speeds i.e., 10 to 40 mm per second at 10N	Adding the chromium to the titanium reduces surface damage & improvises fretting corrosion resistance.
Manuel of Alberto Bortagaray et al. [58].	Titanium dental implants & the abutments	Confocal microscope, artificial salvia. Emerged for three days.	Impactof the electro galvanic corrosion every used material when in contact with the c.p-Ti displayed no statistical vital differences.
Ala’a Al Otaibi et al. [59]	2 same kind of cp titanium implant fixtures, Co-Cr framework	Open-circuit potential, artificial saliva, electrochemical impedance spectroscopy, chronoamperometric and cyclic potential-dynamic polarization.	Intensity of the uniform corrosion rises with immersion time in artificial saliva ranging from 1–24 & then to 48 h.
Alex E. Pozhitkov et al. [60].	Titanium Implants	Sequencing, DNA, Electrochemical measurements, oral microbiota, Spectrometry, Bacterial culture conditions.	Titanium was found in twenty-eight samples of plaque that corresponded to nearly sixteen patients.
Danieli C. Rodrigues et al. [34].	Titanium Implants	Scanning Electron & Digital Microscopy	3D microscopic analysis depicted evidences of corrosion & bulk exposure i.e., post-cleaning
Daniel Olmedo et al. [18].	Orthopedic and Titanium Implants, Grids and Titanium mini-plates	Crystallography, micro-chemical analysis & exfoliative cytology	Whether passivated or noble, every metal shall face slow removal of the ions from surface due to temporal and local variations in environment & micro-structure
Sutow et al. [53].	Cast Co-Cr-Mo, Type 316LVM stainless steel, wrought Co-Cr-W-Ni, Nitrided and Non-nitrided Ti-6Al-4V ELI & CPTi, crevice cell, Grades I & 4	Ringer solution, 37 °C, pH = 7. The Anodic Polarization was performed at the selected levels	The results presented that the treatment of HNO_3_ passivation reduced the Crevice corrosion susceptibility but discoloration and dulling of the CP-Ti was present which recognized 600 mV was excess of O_2_ reduction potential.
Ravnholt and Jensen et al. [39].	Titanium with prosthodontic and amalgam	0.9 percent NaCl Solution Potentiostat	No pH or alteration in pH was detected when cobalt chromium, composite, stainless steel, silver palladium alloys or gold was in the metallic contact along titanium. Alterations took place when amalgam came in contact with the titanium.
Reclaru and Meyer et al. [56].	Silver-palladium, ternary titanium, gold, Co-Cr alloy. Also Ti implant abutment thing	Artificial saliva, scanning potentiostat, Room temperature	The coupling is to have weak anodi polarization in coupling. Current released by galvanic cell should be weak. Crevice potential has to be higher than common potential
Cortada et al. [27].	Titanium oral implant coupled along with various metal superstructures like gold alloy, cast-titanium, silver-palladium alloy, machined-titanium, chromium-nicke l alloy	Artificial saliva at nearly 37 °C. Technique of Coupled plasma mass spectrometry	Titanium oral implant coupled along with chromium-nickel alloy releasing high amount of ions & implant coupled along with titanium superstructure that present low values of the ions released
Aparicio et al. [61].	Pure titanium blasted with various materials	Electrochemical behavior	More surface area due to increase in surface roughness that causes differences in electrochemical behavior & corrosion resistance of blasted CP-Ti
Cyril Sedarat et al. [45].	Titanium alloy NaCl 0.9 percent & human serum, spectrophotometer for atomic absorption	NaCl 0.9 percent & human serum, spectrophotometer for atomic absorption	4 percent toxic V & 6 percent Al may eicit systematic and local reactions or may inhibit differentiation and cellular proliferation
